# Allergological study in patients vaccinated against COVID-19 with suspected allergic reactions

**DOI:** 10.1186/s13223-022-00685-z

**Published:** 2022-05-27

**Authors:** Vicente Jover Cerdá, Ramón Rodríguez Pacheco, Joan Doménech Witek, Sonia Alonso Hernández, Rafael Durán García, Marina Real Panisello, Francisco Manuel Marco de la Calle

**Affiliations:** 1 Allergology Department, General University Hospital of Elda, Ctra. De Sax, s/n – 03600 Elda, Alicante, Spain; 2 Pathologic Anatomy Department, General University Hospital of Elda, Alicante, Spain; 3 Pharmacy Department, General University Hospital of Elda, Alicante, Spain; 4 Clinical Analysis and Immunology, General University Hospital of Alicante, Alicante, Spain

**Keywords:** Anaphylaxis, Basophil activation test, Excipient allergy, Hypersensitivity, Polyethylene glycol, SARS-CoV-2, COVID-19 vaccines

## Abstract

**Background:**

One of the main barriers to vaccination against SARS-CoV-2 is the fear of developing hypersensitivity reactions to any of its components. Although these reactions are very rare, it is necessary to establish an effective protocol to detect patients at risk of developing them. The aim of this study was to evaluate hypersensitivity reactions in vaccinated patients in order to allow or not to complete the vaccination protocol.

**Methods:**

Descriptive and cross-sectional study in which patients with suspected hypersensitivity to SARS-CoV-2 vaccines were evaluated. All patients underwent skin prick test (SPT) and/or intradermal test (IDT) with the vaccines and their excipients. In patients with positive IDT with the vaccine, a histopathological and immunohistochemical study was performed by skin biopsy. A basophil activation test (BAT) and a lymphoblastic transformation test (LTT) were also performed.

**Results:**

Sixteen patients with suspected hypersensitivity to SARS-CoV-2 vaccine (12 received Comirnaty^®^, 3 received Vaxzevria^®^, and 1 received Spikevax^®^) were evaluated. Half had immediate hypersensitivity reactions and half had delayed reactions. All SPTs to excipients and vaccines were negative. IDTs with all excipients were negative. IDTs with vaccines were positive in 11 patients and negative in 5. The histological and immunohistochemical study of the two selected patients with positive IDT with vaccine showed T-lymphocyte involvement. BAT and LTT were negative in both cases. The vaccination protocol could be completed in 7 of 16 patients (44%) studied. The remaining 9 patients did not receive the second dose: 5 because vaccination was not required and 4 because they refused to be vaccinated.

**Conclusions:**

Thanks to the allergological and immunohistochemical study, the vaccination protocol could be completed in about half of the patients who presented suspected hypersensitivity reactions to SARS-CoV-2 vaccines. IDTs with vaccines could be a valuable method for assessing the immunogenicity of the vaccines.

## Background

2021 has become a year of hope for overcoming the pandemic initiated in 2019 by the SARS-CoV-2 coronavirus, thanks to the development of several safe and effective vaccines that have made it possible to reduce mortality, severe infection, and even virus transmission [1, 2]. However, the implementation of the vaccination campaign has been marked by great fear of possible allergic reactions and negationism.

At the time of writing this article, the 4 types of vaccines authorized by the European Medicines Agency (EMA) have been used in Spain. According to the Spanish Agency for Medicines and Health Products (AEMPS), 82,518,671 doses had been administered: 91.7% with one dose or 89.7% with a complete regimen (December 17, 2021). Of the doses administered, 71% corresponded to Comirnaty^®^ (BioNTech/Pfizer), 13% to Vaxzevria^®^ (formerly COVID-19 Vaccine AstraZeneca), 13% to Spikevax^®^ (formerly COVID-19 Vaccine Moderna), and 3% to COVID-19 Vaccine Janssen [3].

Most adverse effects following vaccination are due to a protective immune response induced by the vaccine and not to an allergic reaction [4]. In fact, according to the literature reviewed, cases of immediate hypersensitivity (including anaphylaxis) and/or delayed hypersensitivity to SARS-CoV-2 vaccines are very rare. Even the etiopathogenic mechanism of allergic reactions to these vaccines is not clear. A meta-analysis stated that anaphylaxis with the vaccines against SARS-CoV-2 is a rare event, estimating 7.9 cases per million doses worldwide [5]. According to the latest report of the Center for Disease Control and Prevention (CDC) of the United States, 5.0 and 2.8 cases of anaphylaxis per million doses administered have been reported for patients vaccinated with Comirnaty^®^ and Spikevax^®^, respectively [6]. In Spain, the AEMPS has identified 8 cases (0.52%) with Comirnaty^®^, which represents a rate of 7.2 per million doses administered, mainly in women (88%) and with a median age of 33 years [7]. In 5 of the cases (63%) there was a personal history of urticaria or drug or food allergy. Symptoms started within the first 30 min after vaccination and adrenaline was required in 75% of the cases [7].

In the first weeks after the start of vaccination, immediate hypersensitivity reactions were attributed to the excipients polyethylene glycol (PEG) and/or polysorbate 80 (PS80) used in most vaccines. In addition, it is important to note that both excipients have shown cross-reactivity [8, 9]. However, reactions to these compounds are extremely rare and, if any, are due to non-IgE-mediated mechanisms [10]. On the other hand, diagnoses of hypersensitivity to these polymers remain highly controversial, since skin tests with excipients of SARS-CoV-2 vaccines are not yet standardized and their efficacy, specificity, and sensitivity are questioned [1, 11]. Several institutions, including the European Academy of Allergy and Clinical Immunology (EAACI), suggest performing skin tests with vaccines and their components in patients with suspected allergic reactions after the first dose [12]. There are even authors who recommend intradermal test (IDT) with undiluted vaccine as it is not irritant [13, 14]. Other authors do not recommend it because it may cause systemic reactions [15].

Sellaturay et al. confirmed the first case of anaphylaxis to Comirnaty^®^ by a positive IDT with PS80 [16]. Subsequently, isolated cases of immediate hypersensitivity and/or anaphylaxis by positive skin prick test (SPT) and/or positive IDT with PEG and/or PS80, and positive IDT with diluted and undiluted Comirnaty^®^ have been published [17–21]. Also noteworthy are the 17 cases diagnosed with anaphylaxis to Comirnaty^®^ by positive basophil activation test (BAT) with the vaccine and PEG, and by negative skin tests with the vaccine components [10]. In view of this information, the CDC, the EAACI, and the Spanish Society of Allergy and Clinical Immunology (SEAIC) contraindicate vaccination in patients with a history of immediate allergic reaction to the first dose of vaccine or to any of its excipients, especially to PEG in mRNA-based vaccines (Comirnaty^®^ and Spikevax^®^) and to PS80 in adenovirus-based vaccines (Vaxzevria^®^ and COVID-19 Vaccine Janssen) [6, 22–24].

Vaccination in the Health Department of Elda (Alicante, Spain) started on January 6, 2021. Until September 14, 2021, 282,064 doses were administered, of which 152,130 were the first dose and 129,905 the second dose. The most frequently used vaccine was Comirnaty^®^ (218,950), followed by Vaxzevria^®^ (37,771), Spikevax^®^ (21,265), and COVID-19 Vaccine Janssen (4050) [25].

The objective of this study was to describe and analyze the suspected hypersensitivity reactions in patients vaccinated with the first and/or second dose of SARS-CoV-2 vaccines, as well as to know the cause of these reactions among their components. We also evaluated whether these reactions interfered with the vaccination protocol.

## Methods

### Study design

Descriptive, cross-sectional, and retrospective study in which we evaluated the data from all patients referred from the vaccination centers of the Health Department of Elda (Alicante, Spain) to the Allergology Unit with suspected hypersensitivity to SARS-CoV-2 vaccines, from January 6 to September 30, 2021. Patients who reported symptoms already described as side effects in the technical data sheet of the vaccines were discarded. The data were not evaluated until we obtained ethics committee approval on November 26, 2021.

Primary objective was to evaluate immediate and/or delayed hypersensitivity reactions in patients vaccinated with the first and/or second dose of SARS-CoV-2 vaccines, in order to complete or not the vaccination protocol with the same vaccine or another alternative.

Secondary objectives were to know the clinical profile of vaccinated patients; immunohistochemical study of positive IDTs by skin biopsy; BAT and lymphoblastic transformation test (LTT) in patients with suspected hypersensitivity to the anti-SARS-CoV-2 vaccines; and the effect of allergic reactions on compliance with the vaccination protocol.

### Procedures

An anamnesis was performed on each of the selected patients, recording age, sex, personal history of atopy, type of vaccine administered, immediate and/or delayed symptoms after administration of the first and/or second dose of the vaccine, time interval between the administration of the vaccine and the reaction, treatment used after the reaction, and allergological study.

The skin tests were performed with leftover vaccine vials and with the excipients. For SPT, we used: undiluted vaccine; undiluted PEG300; PEG1500, PEG4000, and PEG6000 at 1% and 10%; Movicol^®^ (PEG3350); Gastrografin^®^ (EDTA and PS80); Trigon Depot^®^ (PS80); Betadine Gel^®^ (Macrogol 400, 4000, and 6000), Ultravist^®^ (trometamol and EDTA); PS80 at 1% and 20%; trometamol in water 1:1; and EDTA at 0.3 mg/ml. For IDT, we used: vaccine undiluted and diluted at 1/100 and 1/10; PEG1500 and PEG4000 at 0.01%; PEG6000 at 1:10,000; Trigon Depot^®^ at 1/10; Ultravist^®^ at 1/10; PS80 at 1/1000 and 1/100; trometamol at 1/10; EDTA at 0.3 mg/ml. Both SPT and IDT with the excipients and the vaccine involved were performed in a stepwise manner from lower to higher concentration of the products, with reading at 30 min and at 24–48 h. Histamine at 0.1% was used as a positive control in SPT and physiological serum at 0.9% as a negative control in SPT and IDT.

In patients with suspected delayed hypersensitivity, skin patch tests were performed with undiluted vaccine, PEG at 4%, and PS80 with reading at 72–96 h. In addition, SPT and IDT were performed in 4 controls with immediate and delayed reading at all dilutions of the vaccines and excipients: 2 unvaccinated atopic patients (negative control in IDT with Comirnaty^®^), 1 atopic patient vaccinated with Comirnaty^®^ without reaction (positive control in IDT with Comirnaty^®^), and 1 healthy unvaccinated patient (negative control in IDT with Vaxzevria^®^).

In patients with positive IDT with diluted and undiluted Comirnaty^®^ vaccine, and in order to clarify whether it was an irritant response, a positive skin test suspicious for type IV hypersensitivity, or a T-cell mediated immune response for protection against SARS-CoV-2, we performed a histopathological and immunohistochemical study by skin biopsy of the IDT with the undiluted vaccine. In addition, BAT and LTT with peripheral blood mononuclear cells were performed.

BAT was performed by a commercial technique (Basotest^®^, Glycotope Biotechnology, Berlin Germany) using heparinized whole blood [9]. Aliquots of blood were incubated with undiluted Comirnaty^®^ and at 4 1/10 dilutions and basophil degranulation was determined through membrane-associated IgE, detecting surface CD63 expression.

LTT was performed to measure the cellular response to the vaccine by determining the proliferation of peripheral blood mononuclear cells by dilution of carboxyfluorescein-succinimidyl ester (CFSE) dye. This assay has been used previously for the analysis of cellular response to T-cell-mediated drug reactions [26]. For proliferation assays with the vaccine, we used the same concentrations as in BAT.

According to the results obtained, in patients in whom hypersensitivity was ruled out, the vaccination protocol was completed, with or without a gradual schedule and premedication, with the second dose of the same vaccine or with an alternative vaccine. Vaccination was discarded in patients with suspected thromboembolic conditions or other disorders and/or severe allergic processes in whom hypersensitivity to the vaccine was demonstrated or in doubt.

### Ethics

The Drug Research Ethics Committee of the General University Hospital of Elda (Alicante, Spain) approved the study on November 26, 2021, with the protocol code VACUNALGUEÑA, VERSION 2.

In compliance with the Declaration of Helsinki and Good Clinical Practice and other regulations about personal data protection, all the information provided in this study was treated in accordance with confidentiality criteria.

Patients were informed about the details of the study for which they gave their consent to participate, according to recently published clinical practice [9, 12, 27].

## Results

A total of 24 patients were referred from the vaccination centers to the Allergology Unit with suspected hypersensitivity to the SARS-CoV-2 vaccines. Of these, 8 were discarded because they were considered to have side effects described in the technical data sheet of the vaccines; all tolerated the second dose without problems. Thus, 16 patients were evaluated: 12 women and 4 men aged between 17 and 76 years (mean 52.6 years) (Table 1). Of the 16 patients studied, 12 received Comirnaty^®^ (10 the first dose and 2 the two doses), 3 received Vaxzevria^®^ (2 the first dose 1 the two doses), and 1 patient received Spikevax^®^ (both doses). Ten of the patients had a history of atopy.

**Table 1:** Clinical characteristics and allergological study of patients with suspected allergic reactions

Patient Nº	Age	Sex	Atopy	Vaccine (dose at which the reaction occurred)	Start and type of reaction (approximate information)	Signs and symptoms	Treatment prescribed	IDT with vaccine^a^	Continuation of the vaccination program
1	40	F	Yes	Comirnaty^®^ (1st dose)	5’ (immediate)	Facial paresthesias with peribucal burning and dizziness	Saline solution	1/100: + 1/10: + Undiluted: +	2nd dose Comirnaty^®^: YES (gradual with premedication)^b^
2	55	F	Yes	Comirnaty^®^ (1st dose)	5’ (immediate)	Pruritus on the palate, dyspnea with 80% SatO_2_, generalized burning sensation, chronic diarrhea (3 months, from 10 days after vaccination)	IV corticoids IV and adrenaline	1/100: + 1/10: + Undiluted: +	2nd dose Comirnaty^®^: NO (refused vaccination)
3	73	M	Yes	Comirnaty^®^ (1st dose)	72 h (Delayed)	Nausea and facial angioedema	IV corticoids and H1A	1/100: − 1/10: + Undiluted: +	2nd dose Comirnaty^®^: YES (gradual with premedication)^b^
4	73	F	Yes	Comirnaty^®^ (1st dose)	30–36 h (Delayed)	Acute generalized urticaria	IV corticoids and H1A	1/100: − 1/10: − Undiluted: −	2nd dose Comirnaty^®^: YES
5	76	F	No	Comirnaty^®^ (1st dose)	36–48 h (Delayed)	Generalized pruritus with dyspnea, abdominal swelling, ankle edema, possible subsegmental pulmonary thromboembolism, mild erythematous rash on arms and neckline	IV corticoids and H1A	1/100: − 1/10: − Undiluted: −	2nd dose Comirnaty^®^: NO (refused vaccination)
6	68	M	No	Comirnaty^®^ (1st dose)	60’ (Immediate)	Wheals on upper limbs	No	1/100: − 1/10: − Undiluted: -	2nd dose Comirnaty^®^: YES
7	29	F	Yes	Comirnaty^®^ (1st dose)	5’ (Immediate)	Generalized erythematous rash with a few papules and facial angioedema, more intense at 24 h	IV corticoids and H1A	1/100: + 1/10: + Undiluted: not done	2nd dose Comirnaty^®^: YES (gradual with premedication)^b^
8	75	M	Yes	Comirnaty^®^ (1st dose)	6 h (Delayed)	Red-vinous macules on lower limbs, upper limbs, thorax and back without itching of 3 weeks of evolution	IM and oral corticoids and H1A	1/100: − 1/10: − Undiluted: −	2nd dose Comirnaty^®^: YES
9	58	F	Yes	Comirnaty^®^ (1st and 2nd dose)	> 24 h (Delayed)	With the 1st dose, local swelling at 24 h and generalized urticaria at 48 h With 2nd dose, maculopapular rash and coldness in lower limbs at 24 h	H1A	1/100: − 1/10: + Undiluted: +	2nd dose Comirnaty^®^: NO (had passed COVID-19 two months before reaction)
10	51	F	No	Comirnaty^®^ (1st dose)	5’ and 24 h (Immediate and delayed)	At 5', erythematous rash on neckline At 24 h, angioedema on the arms and hemiface	IV corticoids and H1A	1/100: + 1/10: + Undiluted: +	2nd dose Comirnaty^®^: YES (gradual with premedication)^b^
11	28	F	Yes	Comirnaty^®^ (2nd dose)	10’ (Immediarte)	Pruritus of the tongue and throat, possible edema of the uvula and swelling of the neck	IV and oral corticoids	1/100: − 1/10: + Undiluted: +	Not necessary (he received the two doses)
12	17	F	No	Comirnaty^®^ (1st dose)	48 h (Delayed)	Wheals on the injection arm, fever and facial angioedema	Oral corticoids and H1A	1/100: − 1/10: + Undiluted: +	2nd dose Comirnaty^®^: NO (had passed COVID-19 one month before reaction)
13	54	F	No	Spikevax^®^ (2nd dose)	12–24 h (Delayed)	Fever, generalized urticaria and arthromyalgia	H1A	1/100: − 1/10: + Undiluted: +	Not necessary (he received the two doses)
14	60	F	No	Vaxzevria^®^ (1st dose)	30’ (Immediate)	At 30', generalized erythematous and pruritic rash, dizziness and bruises on lower limbs At 17 days, post-vaccinal headache	IV corticoids, H1A and adrenaline	1/100: + 1/10: + Undiluted: +	2nd dose Comirnaty^®^: NO (refused vaccination)
15	61	M	Yes	Vaxzevria^®^ (1st and 2nd dose)	40 h (Delayed)	Acute urticaria-angioedema with both doses	IV corticoids and H1A	1/100: − 1/10: − Undiluted: −	Not necessary (he received the two doses)
16	24	F	Yes	Vaxzevria^®^ (1st dose)	10’ and 28 days (Immediate and delayed)	At 10', abdominal pain, stridor and dyspnea At 28 days, dizziness, vomiting, facial paralysis, severe headache, numbness of lower limbs for < 24 h	IV corticoids and adrenaline	1/100: − 1/10: + Undiluted: +	2nd dose Vaxzevria^®^: NO (refused vaccination)

IV: intravenous; IM: intramuscular; H1A: H1-antihistamines; F: female; M: male

^a^The results for the 24 and 48 h readings were the same

^b^Premedication consisted of chlorpheniramine 4 mg and prednisone 30 mg 13 h and 1 h before vaccination

### Hypersensitivity reactions

None of the 16 patients reported previous hypersensitivity reactions with PEG and/or PS80 or with other vaccines, but they had previous reactions with drugs such as penicillins, pyrazolone, or other non-steroidal anti-inflammatory analgesics.

After the administration of the vaccines, 6 patients had immediate hypersensitivity reactions (< 60') and 8 patients had delayed reactions (≥ 6 h). Two of the patients had both immediate and delayed reactions (Table 2). Symptoms were urticaria and/or angioedema, gastrointestinal, neurological, upper respiratory, lower respiratory, and cardiovascular. Most of them were mild to moderate. They were severe in 4 patients (patients nº 2, 5, 14, and 16).

**Table 2:** Results of skin tests with the responsible vaccines according to the type of symptoms

Symptoms	No patients	Skin tests with vaccines			
		SPT +	SPT−	IDT + ^a^	IDT−
Immediate	6	0	6	5	1
Delayed	8	0	8	4	4
Both	2	0	2	2	0
Total	16	0	16	11	5

IDT: intradermal test; SPT: skin prick test

^a^All patients with positive IDT had positive results with the undiluted and 1/10 dilution vaccine. In 5 patients, IDT was positive with the 1/100 dilution. None had a systemic reaction with IDT

These reactions were treated with intravenous corticosteroids and adrenaline in 3 patients, corticosteroids (intravenous, intramuscular or oral) and H1-antihistamines in 9 patients, only H1-antihistamines in 2 patients, and only intravenous and oral corticosteroids in 1 patient. Two patients did not receive any treatment (Table 1).

### Skin tests, histological and immunohistochemical studies

The mean time elapsed between the hypersensitivity reaction and the skin tests was 42 days (7 to 147 days). In 11 patients the study was completed in less than 8 weeks.

**Fig. 1:**
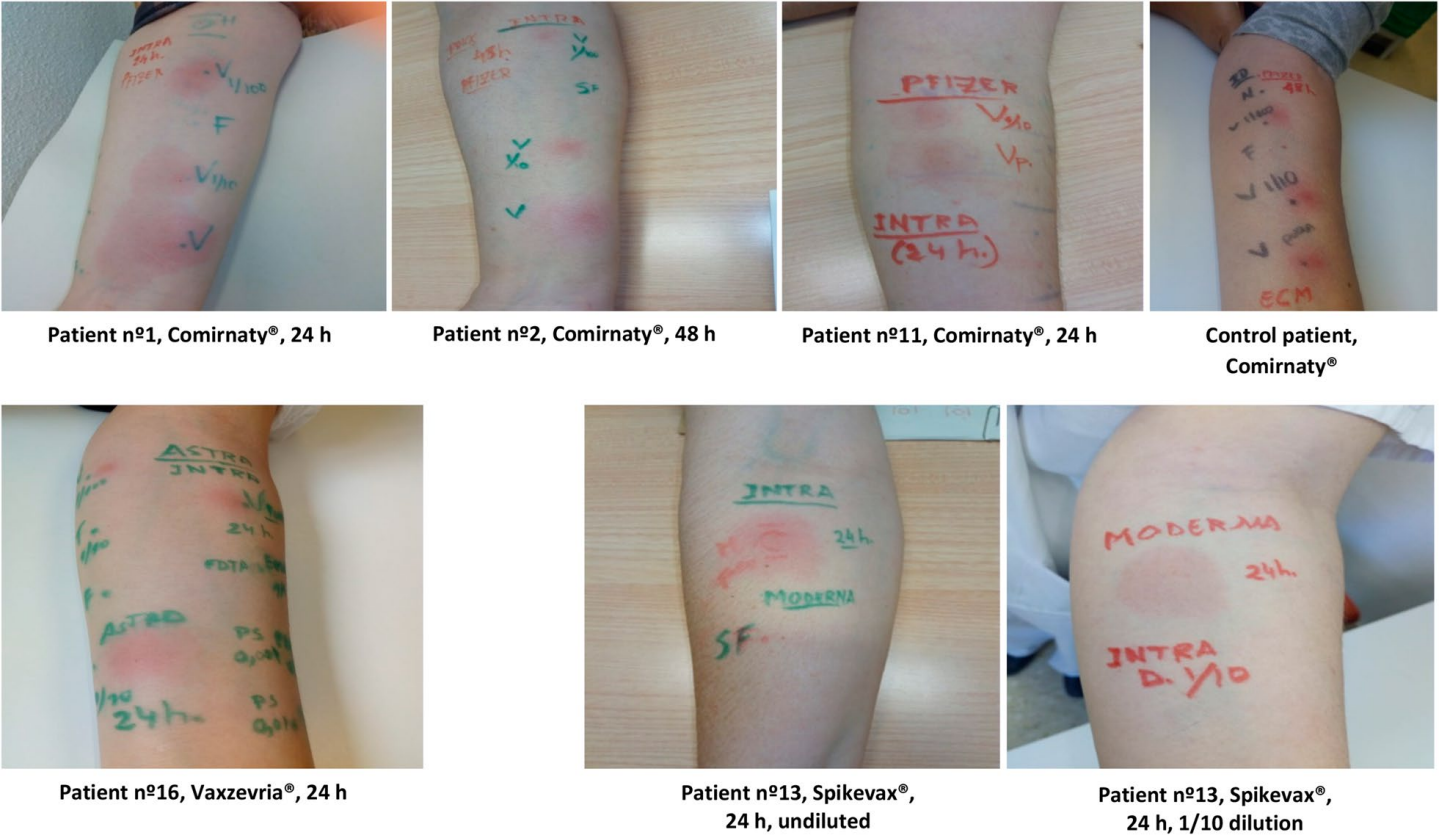
IDT with Comirnaty^®^, Vaxzevria^®^ and Spikevax^®^

All SPT with vaccines and excipients were negative, as well as skin patch tests performed in 7 delayed and 2 immediate reactions. Figure 1 shows some of the IDTs with Comirnaty^®^, Vaxzevria^®^, and Spikevax^®^ and Table 2 shows the results of the skin tests with the responsible vaccine according to symptoms (11 positive IDT and 5 negative IDT). IDTs with all excipients were negative. In the 4 controls, all skin tests with the excipients were also negative.

**Fig. 2:**
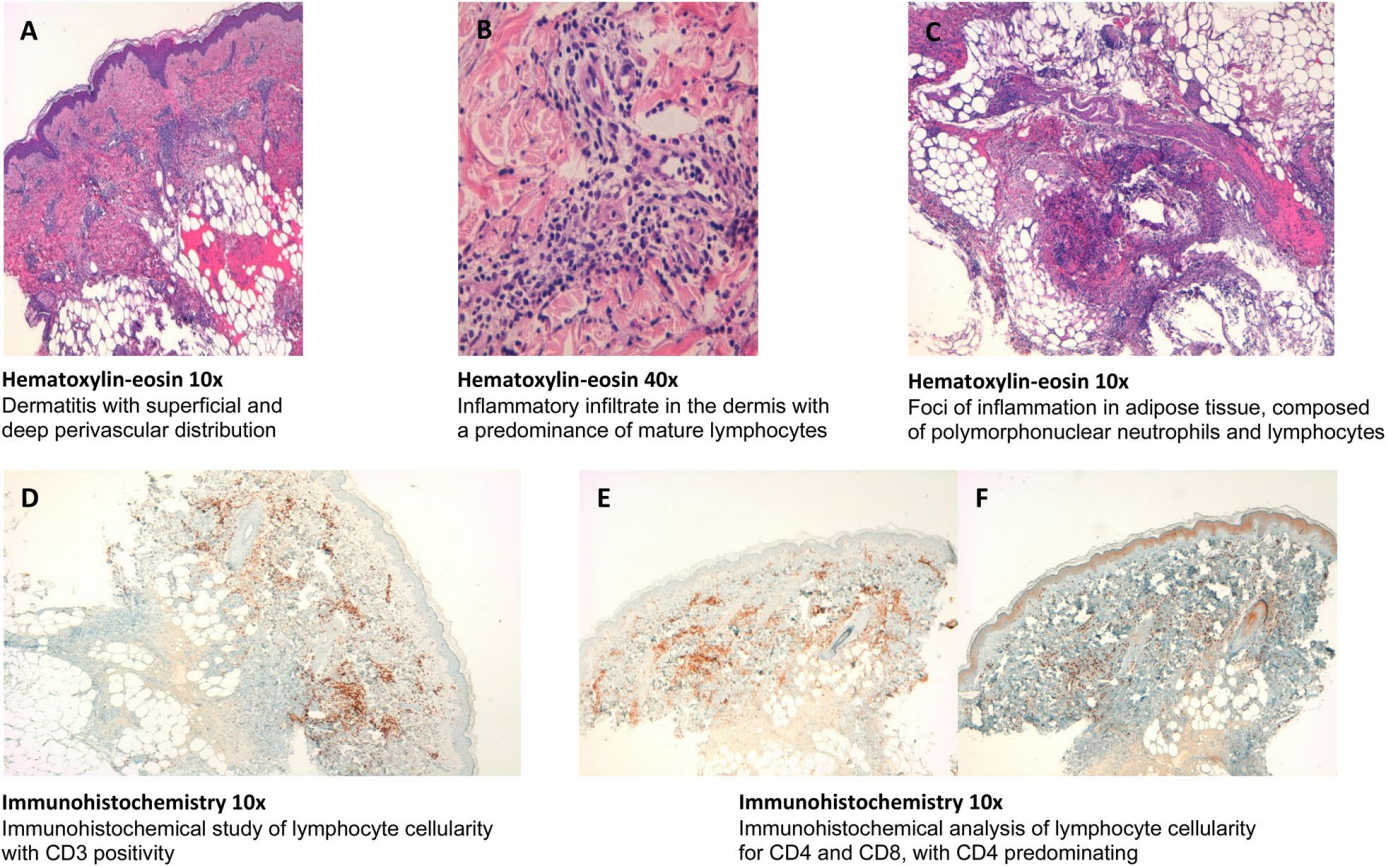
Histological and immunohistochemical study of patient no 1

**Fig. 3:**
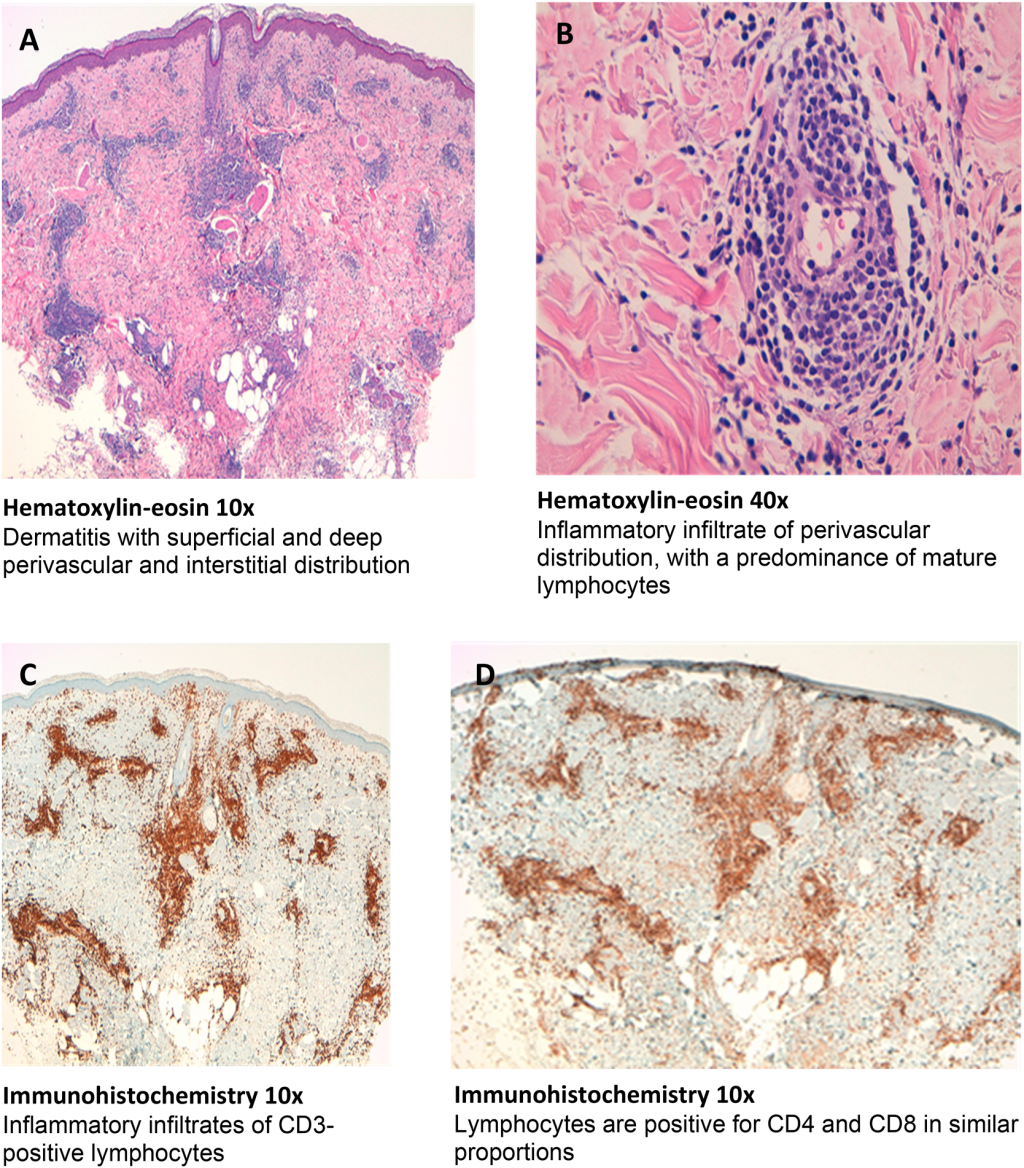
Histological and immunohistochemical study of patient no 2

Histological and immunohistochemical study was performed in two of patients with positive IDT with the vaccines (since it is an invasive test, it was not performed in all of them due to ethical considerations). Histological and immunohistochemical study of positive IDT with undiluted Comirnaty^®^ vaccine was performed in patient nº1 with reading at 24 h (Fig. 2) and at 48 h in patient nº2 (Fig. 3).

Histologic study of patient nº1 showed a mixed inflammatory infiltrate in the dermis, with perivascular and interstitial distribution, consisting predominantly of mature lymphocytes, in addition to some polynuclear neutrophils and isolated eosinophils and mast cells. The epidermis showed a slight vacuolar change of the focal basal layer (Fig. 1A–C). In the histological study of patient nº2, a dermatitis with superficial and deep perivascular and interstitial distribution was observed. The inflammatory infiltrate was predominantly composed of mature lymphocytes with some eosinophils. In the lumen of some capillaries, some polymorphonuclear neutrophils could be recognized. The epidermis presented slight focal vacuolar change of the basal layer with scarce exocytosis of lymphocytes in the stratum basale (Fig. 2A, B). Vasculitis was not seen in both patients. The results of the immunohistochemical study of both patients are shown in Figs. 1D–F, 2C, D.

BAT was negative in both cases to all dilutions of Comirnaty^®^, suggesting the absence of specific IgE to the vaccine components in the basophils of both patients. Likewise, no proliferation of the patients' lymphocytes was observed in response to the vaccine by LTT, so the vaccine components do not appear to be directly responsible for a cellular hypersensitivity reaction.

### Completion of the vaccination protocol

The vaccination protocol was completed in 7 of 16 (46%) patients (6 with Comirnaty^®^ and 1 Spikevax^®^). Four of these patients had positive IDT and received vaccine with a gradual schedule and premedication as a precaution with good tolerance.

The remaining 9 patients did not receive the second dose; 5 because the vaccine was not necessary as they had passed COVID-19 before reaction or they had received the two doses (4 has positive IDT and 1 had negative IDT) and 4 because they refused to be vaccinated due to fear because they had moderate/severe reactions (3 had positive IDT and 1 had negative IDT). Despite having negative IDT with the vaccine, patient nº 5 had a possible subsegmental pulmonary thromboembolism, so the vaccine was not offered. The 4 controls tolerated the corresponding vaccine.

## Discussion

Despite the different variants of SARS-CoV-2 that have been emerging since the beginning of the pandemic, or those that may appear in the near future, mass vaccination of the population is essential to control both the harmful effects and the spread of the virus. However, fear of the occurrence of adverse events, especially hypersensitivity reactions, has triggered mistrust in the population. Although such reactions are rare, it is necessary to establish an effective protocol to detect patients at risk of developing them.

Concurrently to our study, similar studies have also been conducted in other regions of Spain and in other regions of the world with similar results [4, 19, 21, 28–35]. After 282,064 doses administered during the period studied in our Health Department Area, we identified 16 (0.005%) patients with suspected hypersensitivity reaction to the vaccines (0.009% with Vaxzevria^®^, 0.004% with Spikevax^®^, and 0.007% with Comirnaty^®^), similar to that described by other authors with the first dose of Comirnaty^®^ [28, 36, 37]. It is important to highlight that most adverse events occurred in women, with a mean age of 52 years, and a history of previous allergic reactions.

Of the hypersensitivity reactions identified in our study, 6 patients had immediate reactions (4 mild and 2 severe), 8 had delayed reactions (7 mild and 1 severe), and 2 had both immediate and delayed reactions (1 mild and 1 severe), especially cutaneous (urticaria and/or angioedema) and with the first dose of Comirnaty^®^. These findings were similar to those previously described. However, the form of presentation varied from one study to another. Loli-Ausejo et al. reported no anaphylaxis and attributed the symptoms to non-IgE-mediated mechanisms [28]; the CDC reported 83 cases of mild cutaneous or respiratory reactions (0.0044%) and 21 cases of anaphylaxis with Comirnaty^®^ [38]; and Blumenthal et al. described 1.95% of acute allergic reactions and a rate of 2.47 cases of anaphylaxis per 100,000 doses administered [35]. In contrast, Shavit et al. observed that 98% of the 429 subjects who received a dose of Comirnaty^®^ had no immediate allergic events, 1.4% had only minor allergic reactions, and 0.7% had anaphylactic reactions [39]. There are other studies where only delayed reactions were described, due to a delayed type IV hypersensitivity mediated by T-cells [29, 32, 33].

We have observed that SPT with vaccine and SPT and IDT (immediate and delayed reading) with excipients have not been effective in the diagnosis of immediate or delayed hypersensitivity reactions, so that, as other authors believe, skin tests with excipients have very low sensitivity and specificity [1, 11]. Some authors use Refresh Tears^®^ (containing PS80) as a reagent, which has an irritant effect and is therefore not recommended [1, 11], but others believe that SPT with vaccine and excipients can be useful for diagnosis, despite the fact that in some of them the test was negative [28, 36, 37]. Even so, there are isolated published cases of positive SPT with PEG6000 and positive IDT with Comirnaty^®^ 1/100 dilution [21], and positive SPT with PS80 and Comirnaty^®^, without performing IDT [19], even positive SPT with PEG4000 at 1% with associated systemic reaction in which IDT is not recommended [15]. Nevertheless, these studies attempted to complete the vaccination protocol, and the majority of patients tolerated the second doses, either fractionated or with premedication [20, 21]. As in other studies, we have observed that skin patch tests with vaccine and excipients did not contribute to the diagnosis of delayed reactions, so it was recommended to complete the vaccination program, especially in cases of mild exanthema and large local reactions [29, 35].

Regarding IDT with the vaccine, in our study attention was drawn to the 11 patients with positive results (5 with immediate reactions, 4 with delayed reactions, and 2 with both) at 24–48 h with the vaccine diluted at 1/100, specially with the vaccine diluted at 1/10 or undiluted, without presenting associated systemic reaction. Some authors do not perform IDT when the SPT is positive with the vaccine or its excipients and others do not recommend it [15, 19, 28]. According to some studies, IDT with Comirnaty^®^ should not exceed 1/100 dilution to avoid irritant reactions or false positives, or even severe anaphylactic reactions [15, 21]. However, others claim that SPT and IDT with Comirnaty^®^ can be useful at 1/10 dilution and undiluted as they have been shown to be non-irritant in predicting immediate reactions [13, 14]. Bianchi et al. also used IDT with Comirnaty^®^ at 1/1000 and 1/100 dilution with positive results in 6 patients with mucous-cutaneous adverse reactions and in 12 vaccinated volunteers and negative in 6 unvaccinated volunteers, concluding that this may be a sign of cellular immune protection rather than an allergy to the SARS-CoV-2 spike protein or vaccine components [34]. Turner et al. reported that the vaccine is capable of eliciting a delayed intradermal response in vaccinated subjects without PEG allergy [4]. However, further studies are needed to investigate the usefulness of SPT and IDT with vaccines and to clarify the pathological mechanism of IDT reactions.

Interestingly, in contrast to LTT results, intradermal injection of the vaccine in the two patients selected for immunohistochemical study produced a reaction involving T-lymphocytes; CD4 predominance in one case and a mixture of CD4 and CD8 in the other. This in vivo response could be due to the production of SARS-CoV-2 spike protein encoded by the vaccine-containing RNA, by antigen presenting cells of the skin, and by memory T-cells to peptides derived from this protein. Recent reports have described that in previously exposed patients, intradermal injection of recombinant spike protein induces a delayed-type hypersensitivity response involving T-lymphocytes [30, 31]. The negativity of the in vitro cellular response could be due to the inefficiency of the liposomal vaccine construct to induce spike protein expression from the vaccine RNA under the culture conditions used, so that the vaccine components do not appear to be directly responsible for a cellular hypersensitivity reaction. BAT was negative in both cases to all dilutions of the vaccine, suggesting the absence of specific IgE to the vaccine components in the basophils of the two patients. Surprisingly, Warren et al. described 17 patients with anaphylaxis with positive BAT to vaccine and PEG and negative skin tests to vaccine components [10]. The histological and immunohistochemical findings are similar to other published studies with large local reactions after administration of Spikevax^®^, using a single skin biopsy, but which have been considered as T-cell-mediated type IV delayed hypersensitivity reactions [32, 33]. We wonder if in these cases, we are also facing a sign of protective cell-mediated immunity rather than a delayed type IV hypersensitivity reaction, considering that most of them tolerated the second doses [33].

According to the result of the 11 positive IDT and the immunohistochemical study in the 2 selected patients, in which we observed intense lymphocyte activity in the IDT with the undiluted vaccine, in addition to the negative result in LTT and BAT, and based on the bibliographic references consulted on the protective role of cellular immunity [4, 30, 31, 34], we decided to inoculate the second doses or an alternative vaccine in these patients, in order to ensure that the majority of them completed the vaccination protocol.

Despite positive IDT results, patients with mild reactions to the first dose of the vaccine received the second dose without any tolerability problems [11, 40, 41]. Thus, we consider that routine skin testing should not be performed in these patients. On the other hand, patients who have experienced moderate or severe reactions to the first dose should be referred to an allergist for evaluation and skin testing with the suspect vaccine. If the test results negative, the reaction is not considered IgE-mediated and the second dose can be administered with 30 min of observation. If the test results positive, maximum caution should be taken. Administering the second dose in graded doses with premedication under close observation could be an option, although shared decision-making with the patient should be necessary [5, 42].

The main strength of the study is the clinical idea that justifies it, since the objective is to assess the impact of an interventional screening program in high-risk patients. However, due to the small number of patients, who belong to a very specific region, caution should be taken when extrapolating these results to the general population. Therefore, further studies with a larger population and a greater representation of all possible regions are needed.

## Conclusions

Thanks to the allergological and immunohistochemical study, we have been able to complete the vaccination protocol in about half of the patients who presented suspected hypersensitivity reactions. Hence the importance of the figure of the allergist together with other specialties in the resolution of problems regarding hypersensitivity reactions with anti-SARS-CoV-2 vaccines, given that the objective is to ensure that a large majority of the population completes the vaccination protocol. On the other hand, the finding of IDTs with the vaccines themselves could constitute a valuable method for assessing the immunogenicity of the vaccines, although it would be necessary to define the specificity of the response evoked by the inoculum; either some component of the vaccine or the SARS-CoV-2 antigen encoded by the vaccine. Thus, we consider that routine skin testing should not be performed in patients with mild reactions, only in those with moderate and severe reactions.

## Data Availability

Not applicable.

## Abbreviations

AEMPS: Spanish Agency for Medicines and Health Products
BAT: Basophil activation test
CDC: Center for Disease Control and Prevention
EAACI: European Academy of Allergy and Clinical Immunology
EMA: European Medicine Agency
IDT: Intradermal test
LTT: Lymphoblastic transformation test
PEG: Polyethylene glycol
PS80: Polysorbate 80
SEAIC: Spanish Society of Allergy and Clinical Immunology
SPT: Skin prick test
